# Cancer and renal insufficiency results of the BIRMA study

**DOI:** 10.1038/sj.bjc.6605979

**Published:** 2010-11-09

**Authors:** N Janus, V Launay-vacher, E Byloos, J-P Machiels, L Duck, J Kerger, W Wynendaele, J-L Canon, W Lybaert, J Nortier, G Deray, H Wildiers

**Affiliations:** 1 Service ICAR, Department of Nephrology, Pitié-Salpêtrière Hospital, 47-83 Boulevard de l’Hôpital, Paris 75013, France; 2 Department of General Medical Oncology, Multidisciplinary Breast Centre, Leuven 3000, Belgium; 3 Department of Medical Oncology, UCL University Saint-Luc Clinics, Brussels 1200, Belgium; 4 Onco-Hematology Unit, Saint-Pierre Clinic, Ottignies 1340, Belgium; 5 Department of Medical Oncology, UCL University Clinics of Mont-Godinne, Yvoir 5530, Belgium; 6 Department of Medical Oncology, Imelda Hospital, Bonheiden 2820, Belgium; 7 Department of Medical Oncology, Notre Dame Clinic, Charleroi 6000, Belgium; 8 Department of Medical Oncology, AZ Nikolaas Hospital, Sint-Niklaas 9100, Belgium; 9 Department of Nephrology, Erasme Hospital, Brussels 1070, Belgium

**Keywords:** renal insufficiency, anticancer drugs, dose adjustment

## Abstract

**Background:**

Half of anticancer drugs are predominantly excreted in urine. Dosage adjustment in renal insufficiency (RI) is, therefore, a crucial issue. Moreover, patients with abnormal renal function are at high risk for drug-induced nephrotoxicity. The Belgian Renal Insufficiency and Anticancer Medications (BIRMA) study investigated the prevalence of RI in cancer patients, and the profile/dosing of anticancer drugs prescribed.

**Methods:**

Primary end point: to estimate the prevalence of abnormal glomerular filtration rate (GFR; estimated with the abbreviated Modification of Diet in Renal Disease formula) and RI in cancer patient. Secondary end point: to describe the profile of anticancer drugs prescribed (dose reduction/nephrotoxicity). Data were collected for patients presenting at one of the seven Belgian BIRMA centres in March 2006.

**Results:**

A total of 1218 patients were included. The prevalence of elevated SCR (⩾1.2 mg per 100 ml) was 14.9%, but 64.0% had a GFR<90 ml min^−1^ per 1.73 m^2^. In all, 78.6% of treated patients (*n*=1087) were receiving at least one drug needing dosage adjustment and 78.1% received at least one nephrotoxic drug. In all, 56.5% of RI patients receiving chemotherapy requiring dose reduction in case of RI did not receive dose adjustment.

**Conclusions:**

The RI is highly frequent in cancer patients. In all, 80% of the patients receive potentially nephrotoxic drugs and/or for which dosage must be adjusted in RI. Oncologists should check the appropriate dose of chemotherapeutic drugs in relation to renal function before prescribing.

The increased incidence of malignancies in patients with chronic renal failure has been discussed since the middle 1970s (Sutherland *et al*, 1977). One study reported that 188 organ tumours (6.7%) of various pathologies were identified in 2817 patients who had chronic renal failure (Cengiz, 2002). Although it is known to be common in the general population (Jones *et al*, 1998), several studies investigated the incidence of renal insufficiency (RI) among cancer patients. Two studies (Launay-Vacher *et al*, 2004; Dogan *et al*, 2005) observed a high prevalence of RI of ∼33 and 27%, respectively. A total of 50–60% of the 4684 cancer patients in the IRMA-1 study had abnormal renal function (GFR<90), whereas SCR levels were normal in most patients (Launay-Vacher *et al*, 2007). These findings emphasise the high incidence of RI in cancer patients. This is an important issue in clinical practise for the handling of anticancer drugs in those patients. As ∼50% of all anticancer drugs are excreted predominantly in the urine as unchanged drug or active metabolite(s), any reduction in renal clearance can result in accumulation of potentially toxic species and overdosage. The dosage of chemotherapeutic agents used in these patients with RI thus frequently requires dosage reduction to avoid severe toxicities (Lichtman *et al*, 2007). Furthermore, using potentially nephrotoxic anticancer drugs also will require specific monitoring and, when available, specific prevention methods to help reduce the risk for renal toxicity, especially in patients who already have abnormal renal function. In fact, of the patients who received an anticancer drug in the IRMA-1 study, 79.9% received at least one drug that required a dosage adjustment or for which there were no data for use in patients with RI and 80.1% received at least one drug that was nephrotoxic (Launay-Vacher *et al*, 2007).

In this report, we present the results of the Belgian Renal Insufficiency and Anticancer Medications (BIRMA) study, a large, national cohort, multicentric, retrospective study that was conducted to assess the prevalence of RI in cancer patients in Belgium. Specific additional goals (not assessed in IRMA-1) were to describe the profile and dosage of the anticancer drugs prescribed according to the level of renal function, and to assess the relation between renal function and anaemia, previous chemotherapeutic treatments, history of renal disease, and metastases.

## Materials and methods

### Study population

The BIRMA study included a total of 1218 solid cancer patients presenting (either in hospital or as outpatients) in an oncology department in one of the seven participating centres. The study consisted of a retrospective patient data collection on the period of March 2006 and was approved by the local ethical committees. Patients were included regardless of disease pathology, treatment (antineoplastic drugs used/to be used, line of chemotherapy). Patients were excluded if they were aged <18 years, had acute renal failure, a diagnosis of haematological disease or presented with end-stage renal disease that required renal replacement therapy (either haemodialysis or peritoneal dialysis).

### Study design and assessments

The following data were collected for each patient: sex, age, SCR, hemoglobinemia, type of tumour, metastasis (bone and visceral), in/out patients, medical history of kidney disease, nephrectomy, and previous and current anticancer drugs prescribed. Doses of current anticancer drugs were also collected. Estimations of renal function were made by estimating glomerular filtration rate (GFR) with the abbreviated Modification of Diet in Renal Disease (aMDRD) formula (Levey *et al*, 1999).


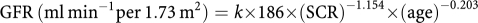
 where *k*=1 (men) or 0.742 (women), GFR indicates glomerular filtration rate, and SCR is measured in mg per 100 ml.

The aMDRD formula was chosen because it is more accurate than Cockcroft–Gault formula (Kleber *et al*, 2007). Renal function was staged in accordance with the clinical practise guidelines published by the Working Group of the National Kidney Foundation (NKF, 2002).

With regard to anticancer therapies prescribed to study patients, the drugs that required dosage adjustment were identified in accordance with their pharmacokinetics and available recommendations from two specific reference books on drug dosage adjustment in patients with RI: *Drug Prescribing in Renal Failure: Dosing Guidelines for Adults, fifth edition* (Aronoff *et al*, 2007) and the *Guide to Prescription Medications for Patients with Renal Insufficiency, third edition* (Launay-Vacher *et al*, 2009).

Then, anticancer medications were classified as ‘yes’ when adjustment was required, ‘no’ when adjustment was not necessary, and ‘no data’ when no data were available in the literature. To obtain profiles of anticancer therapies with regard to renal tolerance, an exhaustive literature search was performed using the National Institutes of Health PubMed database to identify any potential renal side effects of the therapies. If, at least some episodes of nephrotoxicity were identified in the literature search, then the therapy was classified as ‘yes’ which indicated that the drug was ‘potentially nephrotoxic’. Therapies were labelled ‘no’ when no such episodes were identified or when there were no suggestion of potential renal toxicity.

Sub-group analyses on specific patients were performed. Renal function was assessed on patients who had never received any anticancer drugs before this visit (‘chemotherapy-naïve’ patients) and those who had previously received at least one anticancer drug (‘not chemotherapy-naive’ patients).

The link between anaemia and RI was also studied in order to investigate the ‘renal part’ of the anaemia in RI cancer patient.

### End points

The primary end point was to estimate the prevalence of abnormal GFR and RI in all cancer patients presenting in March 2006 in one of the seven participating centres.

The secondary end point was to estimate the frequencies of patients:

- Receiving at least one anticancer drug for which a dose reduction is necessary in case of RI
- Receiving at least one potential nephrotoxic drug.

## Results

### Patient demographics

In total, 1218 patients with various types of cancer were included in the study from seven participating centres. Of these, 64.9% of patients were women, and the mean age of all patients was 61.3 years. The most frequently occurring types of cancer in the study population were breast (510 patients; 41.9%), colorectal (163 patients; 13.4%), lung (95 patients; 7.8%), prostate (91 patients; 7.5%), gynaecologic (79 patients; 6.5%), cerebral (49 patients, 4.0%). Gynaecologic cancer patients included ovarian and uterus cancer patients. Table 1 shows the baseline characteristics of the study cohort.

### RI in BIRMA patients

Among the population, 12.5% had SCR>1.2 mg per 100 ml and 48 patients (3.9%) had a known kidney disease according to the medical records. In spite of this low proportion of patients with elevated SCR and/or diagnosed kidney disease, a majority of patients had in fact a decreased GFR when estimating the GFR with the aMDRD formula: 64.0% had a decreased GFR<90 ml min^−1^ per 1.73 m^2^ (Table 2). Such an estimation of renal function has become the reference method in cancer patients (Kleber *et al*, 2007; Barraclough *et al*, 2008; Holweger *et al*, 2008). Furthermore, when focusing on the patients with a normal SCR (1023 patients), a high prevalence of decreased GFR (665 patients, 65.0%) was also found, meaning that the risk of missing a diagnostic of RI is important if an estimation of the GFR is not performed.

**Figure 1:**
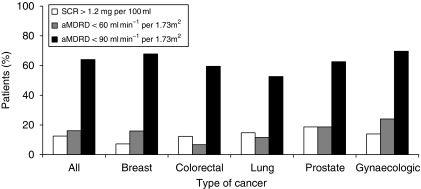
Renal insufficiency for the whole BIRMA population and for the five main types of cancer.

As different tumour types can behave differently, and are treated with different treatment modalities, we also looked at renal function in the different tumour types. In the five main type of cancer: breast, colorectal, lung, prostate, and gynaecologic 67.8, 59.5, 52.6, 62.6, and 69.6% had an aMDRD<90, respectively, (Figure 1) confirming that all cancer patients are at risk for RI whatever the type of cancer.

### Anticancer drug profile

Among the BIRMA population, 1087 patients received an anticancer drug at the time of the visit. These patients received 1852 prescriptions for anticancer drugs, resulting in a mean number of 1.7 drugs per patient.

The prescriptions included 62 different drugs (Table 3). Only a minority of patients received biologicals because these drugs (bevacizumab, sunitinib, sorafenib) were not reimbursed during the study period. Furthermore, many patients received more than one drug (Table 4). Of the 1852 prescriptions, 41.9% were concerning drugs for which a dosage adjustment was necessary. Furthermore, 10.2% of the prescriptions were concerning drugs for which there were no data on their use in patients with RI, meaning that the physicians did not know what to do in case of RI. Finally, 57.3% of the prescribed drugs were potentially nephrotoxic.

Of the 1087 patients, who received an anticancer drug, 78.8% received at least one drug that required a dosage adjustment or for which there were no data on their use in patients with RI, and 78.1% received at least one drug that was potentially nephrotoxic.

Among the 1087 treated patients, 581 patients (52.4%) with a decreased GFR (or no GFR available) received at least one potentially nephrotoxic drug (Table 5). Furthermore, these 581 patients received a total of 709 potential nephrotoxic drugs among a total of 1067 prescriptions (nephrotoxic or not), meaning that some patients received more than one nephrotoxic drug and that 66.4% (709 out of 1067 prescriptions) of the prescribed drugs in this exposed population (581 patients) were potentially nephrotoxic. However, the renal effects resulting of the exposition of these drugs were not investigated.

### Anticancer drug doses

When available, doses were collected for all the patients receiving an anticancer drug at the inclusion visit. In all, 1286 doses were collected for the 1852 prescriptions among the all population. Because GFR<60 is the threshold for many anticancer drugs to consider dose modification, a specific analysis was performed for RI patients receiving chemotherapy for which a dose adjustment may be considered. In all, 161 prescriptions among 147 patients needed a dose reduction according to the level of renal function. Doses were missing for 41 prescriptions (39 patients). Anticancer drugs doses were compared with recommended dosage in RI patients (Launay-Vacher *et al*, 2009). For patients receiving carboplatin, the doses were calculated with the Calvert and Chatelut formulae (Calvert *et al*, 1989; Chatelut *et al*, 1994). Among the prescriptions (120) and patients (108), 46.6–48.3% of the prescription for 50–51.9% of the patients did not have an adequate dose according to their renal function and depending on the formula used to calculate carboplatin doses. 34–44 (28.3–36.7%) prescriptions were overdosed according to renal function. These patients were, therefore, exposed to renal and extra-renal toxicities induced by an overdose. These overdosed prescriptions included: zoledronate (19 prescriptions), carboplatin (6–16 prescriptions according to Chatelut and Calvert formulae, respectively), cisplatin (5), capecitabine (3), and etoposide (1). Furthermore, 32 additional patients without an evaluation of renal function received 36 prescriptions for which a dose adjustment would have been necessary in case of RI.

### Anaemia

Anaemia is common in patients with cancer and is a frequent complication of myelosuppressive chemotherapy (Schwartz, 2007). In all, 55.3% of BIRMA patients presented anaemia (WHO criteria) and 26.9% had a hemoglobinemia<11 g per 100 ml. In all, 6.2% received erythropoiesis-stimulating agents.

Anaemia in cancer patients may be the result of several different etiologies. Particularly, it could result from a direct haematotoxicity of chemotherapy or be of a renal origin in patient with concomitant RI (and cancer disease). In BIRMA patients, we studied the potential effect of RI (GFR<60) on hemoglobinemia and found that RI was a risk factor for anaemia (OR=1.54, (1.11; 2.13)). Furthermore, the anaemia was more frequent in RI patients with a GFR<60 then in patients with a GFR ⩾60. (66.2 *vs* 56.0%, *P*=0.01).

On performing the same analysis of the 302 ‘chemotherapy-naive’ patients, we found that the prevalence of anaemia was still higher in RI patients (77.5 *vs* 59.5%, *P*>0.05), but not significantly (OR=2.04, (0.96; 4.37)). Same trends were found when focusing on the 916 ‘not chemotherapy-naive’ patients, but this time, RI was linked to anaemia in this population (OR=1.5, (1.04; 2.16)) with a prevalence of anaemia higher in RI patients (GFR<60) *vs* in patients without RI (64.5 *vs* 54.8%, respectively, *P*=0.03).

### RI and medical history of chemotherapy

Among the 1218 BIRMA patients, 302 patients (24.8%) had never received any anticancer drugs before this visit (‘chemotherapy-naive’ patients) and 916 (75.2%) patients had previously received at least one anticancer drug (‘not chemotherapy-naïve’ patients; Table 1).

When assessing the prevalence of RI according to the anticancer drug history, ‘not chemotherapy-naive’ patients had a higher prevalence of RI than ‘chemotherapy-naïve’ patients. In all, of 54.3% of the 302 ‘chemotherapy-naive’ patients had a decreased GFR (<90) *vs* 67.1% for ‘not chemotherapy-naive’ patients (*P*<0.0001; Table 2).

### Bone/visceral metastasis

We also investigated the frequencies of RI according to metastasis. The prevalence of decreased GFR (<90) was higher in patients presenting a bone metastasis than in patients without (69.4 *vs* 61.3%, *P*=0.006). This high prevalence was also observed between patients with visceral metastasis and without (69.5 *vs* 60.5%, *P*=0.001).

Three intravenous bisphosphonates were prescribed in BIRMA: ibandronate, pamidronate, and zoledronate. Most patients presenting with bone metastases received zoledronate (220 patients; Table 3). Among them, 67.3% presented a decreased GFR and 50.9% had RI.

### RI and multivariate analysis

As some factors were found to influence renal function in BIRMA cancer patients, two logistic regressions were performed upon the relationship between RI (GFR<90 and GFR<60) and multiple factors (same factors for both analyses). The factors entered into the analysis included gender, age, bone/visceral metastasis, medical history of chemotherapy (Table 6). Age, gender, bone metastasis, and medical history of chemotherapy were found to be risk factors for RI or abnormal GFR.

## Discussion

In this study, we found that RI is highly frequent in cancer patients. However, the prevalence of RI is routinely underestimated in clinical practice when physicians most often base their diagnosis on SCR measurements only. In all, 7% of our patients had no report of SCr determination in their file, but they were probably patients only seen for a routine control (for instance nadir control). It is crucial to outline that SCR is not appropriate for evaluating renal function, but that GFR is calculated by formulas such as aMDRD, also in patients with a normal SCR and even on regular time points in patients coming for a routine consultation without injection of anticancer drugs, because there are still exposed to renal and extra renal toxicity of non-anticancer drugs prescribed for other pathologies. For example, 46.7% of the 120 patients with a GFR<60 and for which drug dosages were available in the medical file received at least one drug with an inappropriately dose according to renal function.

In patients with stage-2 RI, potential drug nephrotoxicity is the main issue. Many studies have demonstrated that pre-existing abnormal renal function is a risk factor for drug-induced nephrotoxicity (Nikolsky *et al*, 2005). As a result, in those patients with mildly decreased renal function, anticancer drugs, antineoplastic, or supportive care, physicians should be aware of the potential risk of nephrotoxicity, and take precautions if possible. If the use of nephrotoxic drug is necessary, it is crucial to adapt the dose, when necessary, according to the renal function and to follow the guidelines for the management of renal toxicity if available, such as for cisplatin (Lichtman *et al*, 2007), for example. The BIRMA patients with a decreased GFR received a mean of 1.2 nephrotoxic anticancer drug. Furthermore, some patients received nephrotoxic associations (Table 4), which expose them to a higher iatrogenic renal risk (cisplatine+gemcitabine, for example). Consequently, it is crucial to avoid (when possible), nephrotoxic combinations of anticancer drugs and non-anticancer drugs. As we did not collect data on other medication, such as pain killers, for which some drugs are clearly nephrotoxic, it is difficult to know how many nephrotoxic drugs (anticancer and others) these patients received in total. We thus may consider that the exposition to all kind of nephrotoxic drug was underestimated in our patients.

The BIRMA was not designed to find the aetiology of RI in cancer patients. However, it provided us with some hypotheses and potential contributing factors. The lower prevalence of RI in ‘chemotherapy-naïve’ patients compared with ‘not chemotherapy-naïve’ patients, of which many are potentially nephrotoxic, suggests a possible role of anticancer drugs. Both univariate and multivariate analysis found that medical history of chemotherapy was a risk factor for abnormal GFR. A causal relationship is difficult to assess based on our data, as other factors (e.g., increased use of pain killers, disease progression with obstructive renal problems…) can contribute as well. Furthermore, patients presenting with bone metastasis had a higher prevalence of RI, but also here the exact causality is difficult to make. It seems plausible that potentially nephrotoxic chemotherapy at least contribute in some part to the declining renal function in cancer patient with progressive disease.

In the BIRMA study, 50.9% of the 220 patients under zoledronate presented a GFR<60 (*vs* 16.1% in the whole population, 22.5% in the 396 bone metastasis patients, 40.0% in the 35 patients with ibandronate, and 66.7% in the 6 patients with pamidronate). It is possible that renal toxicity induced by nephrotoxic anticancer drug, such as zoledronate, adds to the high prevalence of RI, but many other factors can contribute as mentioned previously.

As some factors were not collected in BIRMA patients, such as blood losses, radiation, nutritional deficiencies, inflammation (Schwartz, 2007), it is difficult to exactly quantify the ‘renal part’ of anaemia. Anaemia can have a negative impact on physical and psychosocial function, and quality of life in patients with cancer (Schwartz, 2007), and is a negative prognostic factor (Harper and Littlewood, 2005), so it is important to first diagnose and characterise anaemia, and second, to correct it according to the available guidelines (Aapro and Link, 2008) and to adapt the dose of anticancer drugs in RI patients in order to prevent both renal and extra renal (such as haematoxicity) side effects.

In the BIRMA study, the prevalence of RI was higher than that reported in IRMA-1 study. The prevalence of a decreased GFR (<90) was significantly higher among the Belgian patients than in the French ones (64.0 *vs* 52.9%, *P*<0.0001; Launay-Vacher *et al*, 2007) and in the study conducted by Dogan *et al* (2005). These differences between the prevalence of RI between the three studies may have resulted from differences in patient populations. For example, there were a high proportion of patients with breast cancer in BIRMA study, whereas Dogan *et al* had a high number of patients with gastrointestinal tumours. Furthermore, both Dogan and IRMA-1 studies included younger patients than the patients studied in BIRMA (mean age, 52, 58 and 61 years, respectively).

## Conclusion

Many anticancer drugs can cause RI, and many anticancer drugs require dose adaptation in RI. It may not be always possible not to use potentially nephrotoxic drugs (Launay-Vacher *et al*, 2008). However, it remains very important to be aware of the renal function of patients who receive such drugs, and to monitor renal function on a regular basis, before each course of therapy during treatment.

Our study shows that a significant number of RI patients receiving chemotherapy requiring dose reduction in case of RI did not receive dose adjustment. Oncologists should check the appropriate dose of chemotherapeutic drugs in relation to actual renal function before prescribing to their patients.

Furthermore, in those patients who require nephrotoxic anticancer drugs, and especially those with baseline decreased renal function, cautious selection, and analysis of concomitant drugs should be performed. For example, non-steroidal anti-inflammatory drugs, if possible, should be avoided, as they may increase the renal toxicity of chemotherapy.

## Figures and Tables

**Table 1:** Characteristics of the BIRMA cancer patients at inclusion (March 2006)

	**General population (*n*=1218)**	**Chemotherapy-naïve patients (*n*=302)**	**Not chemotherapy-naïve patients (*n*=916)**	**Comparison: chemotherapy-naïve *vs* not chemotherapy-naïve**
**Variables**	**Mean±s.d.**	**Mean±s.d.**	**Mean±s.d.**	***P*-value**
Age (years)	61.3±12.8	63.0±12.5	60.7±12.8	0.007
Weight (kg)	69.6±14.8	69.6±15.7	69.6±14.5	>0.05
Height (cm)	166.1±9.1	167.5±9.0	165.7±9.1	0.01
Creatininemia (mg per 100 ml)	0.94±0.52	0.96±0.74	0.92±0.42	>0.05
aMDRD (ml min^−1^ per 1.73 m^2^)	82.7±35.9	85.4±26.4	81.7±38.6	0.05
Hemoglobinemia (g per 100 ml)	11.9±1.7	11.9±1.7	11.9±1.7	>0.05
Hematocrit (%)	35.8±4.9	35.3±4.9	36.0±4.9	>0.05
				
	**Number of patients (%)**	**Number of patients (%)**	**Number of patients (%)**	***P*-value**
Bone metastasis	396 (32.5)	36 (11.9)	360 (39.3)	<0.0001
Visceral metastasis	463 (38.0)	87 (28.8)	376 (41.1)	0.0001
Known kidney disease	48 (3.9)	20 (6.6)	28 (3.1)	0.006
Nephrectomy	22 (1.8)	3 (1.0)	19 (2.1)	>0.05

Abbreviations: aMDRD=abbreviated Modification of Diet in Renal Disease formula; BIRMA=Belgian Renal Insufficiency and Anticancer Medications.

**Table 2:** Renal insufficiency among the BIRMA cancer patients according to the K/DOQI, KDIGO classification (NKF, 2002; Levey *et al*, 2005)

**aMDRD–GFR (ml min^−1^ per 1.73 m^2^)**	**General population (*n*=1218)**	**Chemotherapy-naïve patients (*n*=302)**	**Not chemotherapy-naïve patients (*n*=916)**	**Comparison: chemotherapy-naïve *vs* not chemotherapy-naïve**
⩾90, *n* (%)	358 (29.4)	122 (40.4)	236 (25.8)	<0.0001
89–60, *n* (%)	583 (47.9)	122 (40.4)	461 (50.3)	0.002
59–30, *n* (%)	182 (14.9)	37 (12.3)	145 (15.8)	>0.05
29–15, *n* (%)	11 (0.9)	3 (1.0)	8 (0.9)	>0.05
<15, *n* (%)	3 (0.3)	2 (0.7)	1 (0.1)	>0.05
No SCR available, *n* (%)	81 (6.7)	16 (5.3)	65 (7.1)	>0.05

Abbreviations: aMDRD=abbreviated Modification of Diet in Renal Disease formula; BIRMA=Belgian Renal Insufficiency and Anticancer Medications; GFR=glomerular filtration rate; K/DOQI=Kidney Disease Outcomes Quality Initiative; KIDGO=Kidney Disease: Improving Global Outcomes.

**Table 3:** Anticancer drugs most often prescribed to patients in the study

**INN**	**No. of prescriptions**	**Percent of prescription (%)**	**Need for dosage adjustment in RI**	**Potential nephrotoxicity**
Fluorouracil	248	13.6	No	No
Zoledronate	220	12.1	Yes	Yes
Docetaxel	114	6.2	Yes	No
Cyclophosphamide	101	5.5	Yes	No
Epirubicin	94	5.2	No	SC
Trastuzumab	92	5.0	ND	SC
Gemcitabine	90	4.9	No	Yes
Cisplatin	89	4.9	Yes	Yes
Carboplatin	70	3.8	Yes	Yes
Paclitaxel	63	3.5	No	SC
Oxaliplatin	61	3.3	No	Yes
Irinotecan	53	2.9	No	Yes
Doxorubicin	52	2.8	No	SC
Capecitabin	47	2.6	Yes	No
Letrozole	42	2.3	No	No
Tamoxifen	41	2.2	No	SC
Ibandronate	35	1.9	Yes	No
Vinorelbine	32	1.8	Yes	No
Exemestane	30	1.6	No	No
Temozolomide	22	1.2	ND	No
Cetuximab	18	1.0	No	Yes
				
Other	238	12.9	Yes: 28.2%	Yes: 100 prescriptions
			ND: 31.5%	ND: 18 prescriptions
			No: 40.3%	No: 100 prescriptions
				
Total	1852	100	Yes: 41.8%	Yes: 1043 prescriptions
			ND: 10.2%	ND: 18 prescriptions
			No: 47.9%	No: 791 prescriptions

Abbreviations: INN=international non-proprietary name; ND=no data available in the literature for use in patient with RI or for nephrotoxicity; RI=renal insufficiency; SC=sparse cases.

Other: anticancer drugs that were prescribed in <1% of patients in the study (decreasing number of prescriptions): bevacizumab, gosereline, etoposide, fulvestrant, methotrexate, topotecan, anastrozole, dacarbazine, vinblastine, erlotinib, vinflunine, mitoxantrone, bicalutamide, mitomycine, megestrole, pamidronate, imatinib, ifosfamide, vaccine, bleomycin, lomustin, estramustin, fotemustin, gefitinib, leuproreline, octreotide, hydroxycarbamide, pemetrexed, triptoreline, sunitinib, panitumumab, thyrixine, cyproterone, carmustine, rituximab, streptozocine, matuzumab, sorafenib, lanreotide, ipilimumab.

**Table 4:** Anticancer drugs associations most often prescribed to patients in the study

**Main anticancer drugs associations**	**No. of prescriptions**	**Percent of prescription (%)**	**Need for dosage adjustment in RI**	**Potential nephrotoxicity**
Cyclophosphamide–epirubicin–fluorouracil	80	7.4	Yes–No–No	No–SC–No
Fluorouracil–oxaliplatine	53	4.9	No–No	No–Yes
Fluorouracil–irinotecan	36	3.3	No–No	No–Yes
Carboplatin–paclitaxel	27	2.5	Yes–No	Yes–SC
Letrozole–zoledronate	26	2.4	No–Yes	No–Yes
Tamoxifen–zoledronate	23	2.1	No–Yes	SC–Yes
Cisplatine–gemcitabine	21	1.9	Yes–No	Yes–Yes
Cisplatine–fluorouracil	20	1.8	Yes–No	Yes–No
Exemestane–zoledronate	16	1.5	No–Yes	No–Yes
Docetaxel–zoledronate	14	1.3	Yes–Yes	No–Yes

Abbreviations: RI=renal insufficiency; SC=sparse cases.

**Table 5:** Profile of anticancer drugs (chemotherapy, support treatments…) according to the renal function

	**Number of patients with at least one drug: (*n=1087*)**	
**aMDRD–GFR**	**Which is potentially nephrotoxic**	**For which a dose adjustment is required**
**(ml min^−1^ per 1.73 m^2^)**	**Labelled ‘yes’ or ‘ND’**	**Labelled ‘yes’ or ‘ND’**
⩾90, *n* (%)	268 (24.7)	253 (23.3)
89–60, *n* (%)	408 (37.5)	430 (39.6)
59–30, *n* (%)	120 (11.0)	122 (11.2)
29–15, *n* (%)	6 (0.6)	5 (0.5)
<15, *n* (%)	0 (0.0)	0 (0.0)
No Data, *n* (%)	47 (4.3)	44 (4.1)

Abbreviations: aMDRD=abbreviated Modification of Diet in Renal Disease formula; GFR=glomerular filtration rate; ND=no data available.

**Table 6:** Predictions of abnormal GFR and of renal insufficiency (multivariate analysis)

**Variables**	**Odds ratio, (CI_95%_)**	***P*-value**
*Predictions of abnormal GFR (GFR<90 ml min*^*−1*^ *per 1.73 m*^*2*^*)*		
Gender	2.20 (1.65, 2.91)	*P*<0.0001
Age	1.06 (1.04, 1.07)	*P*<0.0001
Bone metastasis	1.12 (0.90, 1.43)	*P*>0.05
Visceral metastasis	0.84 (0.62, 1.14)	*P*>0.05
Medical history of chemotherapy	2.09 (1.53, 2.86)	*P*=0.001
		
*Predictions of renal insufficiency (GFR<60 ml min*^*−1*^ per *1.73 m*^*2*^*)*		
Gender	1.73 (1.21, 2.47)	*P*=0.003
Age	1.08 (1.06, 1.10)	*P*<0.0001
Bone metastasis	1.46 (1.03, 2.6)	*P*=0.04
Visceral metastasis	0.89 (0.63, 1.24)	*P*>0.05
Medical history of chemotherapy	1.36 (0.94, 2.11)	*P*>0.05

Abbreviations: CI=confidence interval; GFR=glomerular filtration rate.
